# A 6D Object Pose Estimation Algorithm for Autonomous Docking with Improved Maximal Cliques

**DOI:** 10.3390/s25010283

**Published:** 2025-01-06

**Authors:** Zhenqi Han, Lizhuang Liu

**Affiliations:** 1 School of Information Science and Technology, Fudan University, Shanghai 200438, China; 2 Shanghai Advanced Research Institute, Chinese Academy of Sciences, Shanghai 201210, China

**Keywords:** maximal cliques, pose estimation, autonomous docking, spatial compatibility, graph filtering

## Abstract

Accurate 6D object pose estimation is critical for autonomous docking. To address the inefficiencies and inaccuracies associated with maximal cliques-based pose estimation methods, we propose a fast 6D pose estimation algorithm that integrates feature space and space compatibility constraints. The algorithm reduces the graph size by employing Laplacian filtering to resample high-frequency signal nodes. Then, the truncated Chamfer distance derived from fusion features and spatial compatibility constraints is used to evaluate the accuracy of candidate pose alignment between source and reference point clouds, and the optimal pose transformation matrix is selected for 6D pose estimation. Finally, a point-to-plane ICP algorithm is applied to refine the 6D pose estimation for autonomous docking. Experimental results demonstrate that the proposed algorithm achieves recall rates of 94.5%, 62.2%, and 99.1% on the 3DMatch, 3DLoMatch, and KITTI datasets, respectively. On the autonomous docking dataset, the algorithm yields rotation and localization errors of 0.96° and 5.82 cm, respectively, outperforming existing methods and validating the effectiveness of our approach.

## 1. Introduction

Autonomous docking systems employ automated guided vehicles (AGVs) or robotic arms to connect one target to another at a specific position and orientation. These systems are extensively utilized in fields such as aerospace [1], weapon mounting [2], industrial assembly [3], and medical services [4], among others. A key step in realizing autonomous docking is the accurate, reliable, and cost-effective estimation of relative pose, which directly influences the quality and efficiency of docking tasks. Traditional docking methods depend on manual alignment, which is often constrained by the operator’s experience and skill, leading to low docking accuracy, slow installation speeds, and inefficiencies. Intelligent autonomous docking systems are crucial for advancing smart assembly and industrial automation.

The advancement of 3D imaging technologies has facilitated point cloud data acquisition, providing rich geometric information about objects. This is especially important for pose estimation in scenarios where color and texture are absent. As a result, methods for pose estimation based on point cloud data have rapidly advanced. The goal of pose estimation is to determine the six degrees of freedom (6DoF or 6D) pose of objects, including 3D orientation and displacement. By calculating the 6D pose of the coordinate system of two objects relative to the 3D imaging sensor, the 6D relative pose between the objects is obtained. Therefore, accurate and robust 6D object pose estimation methods are essential for achieving high precision in relative pose estimation. At present, 6D pose estimation has become a key research area, with the primary approaches being learning-based and geometry-based methods. Learning-based methods [5,6,7] typically use RGBD data and apply neural networks to directly estimate the 6D object pose in the sensor’s coordinate system in an end-to-end manner. While these methods are highly accurate, they require large-scale annotated datasets and lack generalization, making it difficult to apply them across different datasets. In contrast, geometry-based methods offer significant advantages in practical applications because they do not require training data and exhibit strong generalization capabilities.

Geometry-based methods generally rely on 3D point cloud templates, finding at least three pairs of correspondences between the template’s and the scene’s point clouds. The 6D pose is subsequently calculated using singular value decomposition (SVD) or least squares methods. The iterative closest point (ICP) algorithm [8] and its variants [9,10,11,12] are commonly employed for pose estimation. ICP-based algorithms iteratively match point pairs and minimize the distance between them to estimate the 6D pose. However, these methods are sensitive to the initial pose, lack robustness, and are prone to local minima. Feature-based 3D point cloud pose estimation methods have gained considerable attention for their improved robustness. These methods typically involve four steps: local feature extraction, feature matching, outlier removal, and pose solving. Feature extraction can employ either hand-crafted 3D descriptors or learned feature descriptors. Common point cloud descriptors include PFH [13], FPFH [14], PPF [15], FCGF [16], and PREDATOR [17]. However, due to factors such as point cloud noise and the limited discriminative power of local descriptors, significant mismatches and outliers often arise during feature matching. These outliers significantly impact pose estimation accuracy and present considerable challenges to 6D pose estimation.

Dealing with outliers in 3D point cloud correspondence is a critical step in 6D pose estimation. One of the most commonly used methods for this task is the Random Sample Consensus (RANSAC) [18], which employs random sampling to identify consistent point clouds, offering simplicity and high robustness. However, when the proportion of outliers increases, RANSAC requires numerous iterations to identify consistent inliers, making its performance highly susceptible to inefficiency and instability. Spatial compatibility (SC) [19,20,21] is a widely adopted similarity measure in rigid body transformations, known for its robustness and efficiency. SC assumes that the spatial distances of matching points from correct correspondences are approximately the same in both point clouds, while the spatial distances of points from abnormal correspondences differ significantly. Resampling based on SC increases the proportion of inliers, but when the distance between abnormal correspondences is small, some outliers remain undetected. To address this issue, second-order spatial compatibility [22] has been proposed, which reduces the likelihood of abnormal correspondences being included in the resampling set. In recent years, point cloud registration methods based on compatibility graphs [21,23,24,25] have gained widespread attention. These methods utilize graph cliques to globally search for correspondences with high spatial compatibility, reducing outliers within the clique nodes. Yang et al. [23,25] proposed a point cloud registration method based on maximal cliques (MAC), relaxing the constraints of the maximal clique and extracting additional local consensus information. This approach improves the accuracy and robustness of pose estimation in point cloud registration.

However, in the maximal cliques-based pose estimation framework, the time complexity of clique search is $O\left(3^{n/3}\right)$, which is exponential and depends on both the size of the graph and the number of cliques. Therefore, as the number of correspondences increases, the clique search process becomes computationally expensive. Moreover, existing model evaluation methods typically rely on the inlier ratio, which estimates the number of correspondences with alignment errors below a threshold after aligning the hypothetical point clouds. However, due to high outliers caused by feature descriptor limitations or low overlap, the number of inliers is often minimal, making it difficult to accurately identify the optimal pose.

To address these challenges in the maximal cliques-based pose estimation framework [23], such as the time-consuming graph search and the limitations of model evaluation methods, this paper proposes an efficient maximal cliques pose estimation algorithm that incorporates both feature and spatial compatibility constraints. This approach is specifically designed for autonomous docking applications. Specifically, the algorithm first resamples high-frequency signal nodes in the graph using Laplacian filtering, which reduces the graph size and improves computational efficiency. Additionally, the Chamfer distance, which integrates both feature and spatial compatibility constraints, is used to evaluate the alignment accuracy of candidate poses between source and reference point clouds. The optimal 6D pose transformation matrix is then selected based on this evaluation. Finally, a relative 6D pose estimation framework for autonomous docking is proposed, employing a coarse-to-fine strategy: an initial rough 6D pose estimation is performed using the proposed maximal cliques-based algorithm, followed by fine pose refinement with a point-to-plane ICP algorithm.

The main contributions of this paper are as follows:

(1) A resampling method for point cloud matching pairs based on Laplacian filtering is proposed. This method not only increases the proportion of inliers in the resampled matching pairs but also reduces the scale of the graph search, thus improving pose estimation efficiency.
(2) In maximal cliques-based pose estimation methods, a new hypothesis evaluation method is introduced, which considers not only the inlier ratio but also incorporates feature and spatial consistency constraints. This enhances the algorithm’s adaptability to sets of correspondences with high outlier rates.
(3) A relative pose estimation framework for the autonomous docking is proposed. By combining the improved maximal cliques pose estimation algorithm with the point-to-plane ICP algorithm, a coarse-to-fine pose estimation process is achieved and verified using real autonomous docking data.

The remainder of this paper is organized as follows: Section 2 defines the problem and presents the 6D pose estimation with maximal cliques. Section 3 outlines the improved maximal cliques for the 6D pose estimation algorithm. Section 4 presents a relative 6D pose estimation framework for autonomous docking. Section 5 presents an extensive experimental evaluation of the feasibility and effectiveness of the proposed method. Section 6 concludes the paper.

## 2. Problem Definition and MAC

### 2.1. Problem Definition

**Figure 1:**
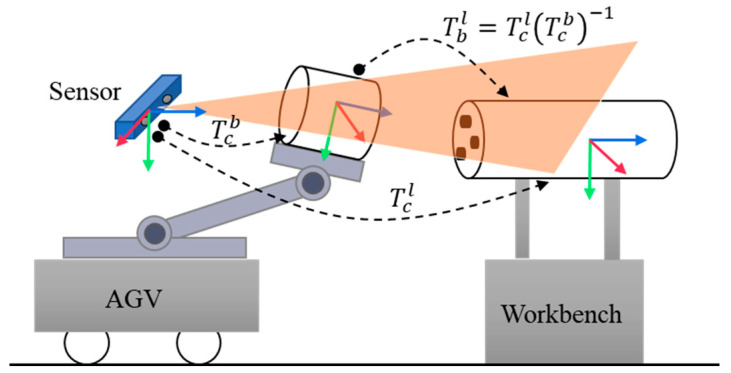
Diagram of automated docking.

In the typical docking scenario shown in Figure 1, an AGV is tasked with docking and assembling the onboard target with the target on the workbench. A 3D imaging sensor is installed in the scene to observe and measure both the vehicle and workbench targets. Since the docking environment is unstructured and the relative position between the 3D imaging sensor and the vehicle is unknown, this scenario differs from typical eye-to-hand or eye-in-hand robotic docking systems.

Assuming the 3D sensor has collected point cloud data for both the vehicle target and the workbench target, and that the 3D point cloud templates for both targets are known, the 6D pose of each target is estimated using a 6D pose estimation algorithm. Specifically, the 6D pose of the vehicle target, $T_{c}^{b}$, and the 6D pose of the workbench target, $T_{c}^{l}$, are determined. The relative 6D pose of the vehicle target with respect to the workbench target is then calculated, using the coordinate system of the workbench target as the global reference.

The 6D pose estimation problem for a target involves finding the optimal transformation matrix between the source point clouds $P=\left\{p_{i}\in R^{3}|i=1,\cdots n\right\}$ and the reference template $Q=\left\{q_{i}\in R^{3}|i=1,\cdots m\right\}$, such that point clouds P aligns correctly with point clouds Q after transformation. Typically, local features are first extracted from the point cloud, and then initial geometric correspondences, denoted as $C_{initial}=\left\{\left(p_{i},q_{i}\right)\in R^{6}|i=1,\cdots ,n\right\}$, are established based on feature matching. Next, a 6D pose estimation algorithm is applied to compute the optimal transformation matrix from the initial correspondences, enabling precise alignment of the transformed point clouds with the reference point clouds.

### 2.2. Maximal Cliques for 6D Pose Estimation

**Figure 2:**
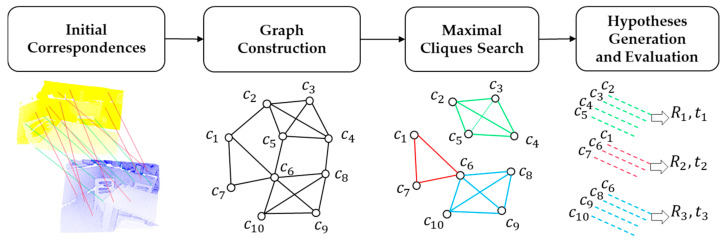
Pipeline of MAC.

Maximal cliques (MAC) registration [23] is a geometric point cloud registration technique that computes the optimal pose matrix by aligning two sets of point clouds. When one point cloud is treated as the reference template and the other as sensor data collected, this method can also be applied for 6D pose estimation. The core idea behind MAC is to model the initial correspondences as a second-order spatial compatibility graph. During graph search, the method employs a maximal clique search to extract more local consensus information, thereby generating accurate pose estimates. The MAC pose estimation procedure consists of four steps: graph construction, maximal cliques search, pose hypothesis generation, and pose hypothesis evaluation. The overall process is illustrated in Figure 2.

#### 2.2.1. Construction of Second-Order Spatial Compatibility Graph

**Figure 3:**
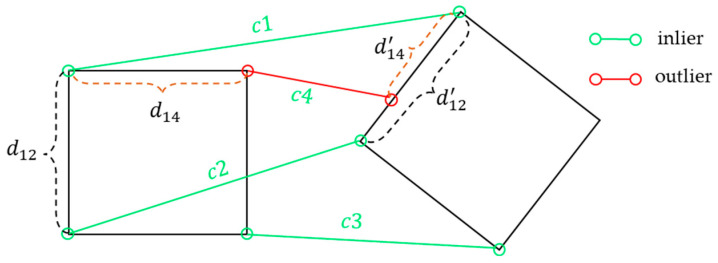
Spatial compatibility.

In the initial correspondence set $C_{initial}$, there are often many mismatched points, i.e., outliers. If these outliers are not filtered out, they can significantly affect the accuracy of pose estimation. In rigid transformations, objects undergo only rotation and translation, with their size remaining unchanged. Therefore, the spatial compatibility assumes that the spatial distance between two corresponding points in the two point clouds is small, indicating a higher correspondence score, as shown in Figure 3. For example, if both c1 and c2 are inlier correspondences, their spatial distances $d_{12}$ and $d_{12}^{\prime }$ in the two point clouds are similar. However, if c1 and c4 are a pair consisting of an inlier and an outlier, their spatial distances $d_{14}$ and $d_{14}^{\prime }$ in the two point clouds exhibit a significant difference. Based on the spatial compatibility constraint, resampling the initial correspondence set can increase the probability of inliers, thereby improving the robustness and efficiency of rigid transformation estimation.

Compared to Euclidean space, graph space more accurately represents the affinity between matching points. Therefore, MAC constructs the initial correspondence set as a second-order spatial compatibility graph, with its adjacency matrix S2C defined as follows:

(1) $$S2C=\left(SC\times SC\right)⊙SC$$

where × represents matrix product and ⊙ denotes the Hadamard (element-wise) product. SC is the first-order spatial compatibility matrix, which is defined as

(2) $$SC_{ij}=\left\{\begin{array}{c} exp\left(-\frac{D{\left(p_{i},q_{i}\right)}^{2}}{2\sigma^{2}}\right),\;if\;D\left(p_{i},q_{i}\right)<\epsilon \;\\ 0,\;\;\;\;\;\;\;\;\;\;\;\;\;\;\;\;if\;D\left(p_{i},q_{i}\right)\geq \epsilon \;or\;i=j \end{array}\right.$$

where ϵ is the distance threshold, and $D\left(p_{i},q_{i}\right)$ is defined as

(3) $$D\left(p_{i},q_{i}\right)=\left\|p_{i}-p_{j}\right\|-\left\|q_{i}-q_{j}\right\|$$

Consequently, the second-order spatial compatibility matrix serves as the adjacency matrix of the graph, where nodes represent matching relationships and edges represent the consistency of these correspondences. Unlike the first-order spatial compatibility matrix, the second-order matrix is more precise in capturing the compatibility between adjacent nodes and is sparser, which accelerates the clique search process.

#### 2.2.2. Maximal Clique Search

A maximal clique in an undirected graph is a subgraph in which an edge exists between every pair of vertices. In the context of point cloud registration, inliers generally exhibit high spatial consistency and tend to cluster within the graph. The MAC method loosens the constraint on the maximal clique, shifting from a focus on global consensus to extracting local graph information. The Bron–Kerbosch algorithm [26] from the igraph library is used to perform the maximal clique search efficiently.

#### 2.2.3. Pose Hypothesis Generation

The maximal clique search identifies a set of matching pairs with high spatial consistency. The pose transformation matrix for each set of matching relations can be determined using the weighted singular value decomposition (SVD) method. The process of calculating the pose transformation matrix is as follows:

(1) Calculate the weighted center of the matched pairs, as given by

(4) $$\bar{p}=\frac{\sum_{i}w_{i}p_{i}}{\sum_{i}w_{i}},\;\bar{q}=\frac{\sum_{i}w_{i}q_{i}}{\sum_{i}w_{i}}$$

(2) Calculate the weighted covariance matrix:
  (5) $$S=\sum_{i}{\left(p_{i}-\bar{p}\right)}^{T}W\left(q_{i}-\bar{q}\right)\;$$
  where $W=diag\left(w_{1},w_{2},\cdots w_{n}\right)$.

(3) Decompose the weighted covariance matrix using SVD to obtain the rotation matrix R and translation matrix t:
  (6) $$S=U\Sigma V^{T}$$
  (7) $$R={VU}^{T}$$
  (8) $$t=\bar{q}-R\bar{p}\;$$

#### 2.2.4. Pose Hypothesis Evaluation

The MAC method uses the number of inliers as the criterion for selecting the best pose transformation. A point is classified as an inlier if, after applying the transformation matrix to the origin cloud, the Euclidean distance between the transformed point and the corresponding reference point cloud is less than a predefined threshold. Among the candidate transformations in the MAC method, the optimal 6D pose matrix (comprising the rotation and translation matrices) is chosen based on the maximum number of inliers that satisfy the predefined error threshold. The optimal transformation matrix achieves the highest number of inliers while adhering to rotation and translation constraints. The evaluation criterion is given by

(9) $$R^{*},t^{*}=\sum I\left(Rp_{i}+t-q_{i}<\epsilon \right)\;$$

where I is an indicator function. It returns a value of 1 when the condition inside the function is true, and 0 when the condition is false. In the experiment, we maintained consistency in the MAC parameter settings, with the predefined threshold value ϵ=0.1 in 3DMatch and 3DLomatch dataset, while ϵ=0.6 in KITTI dataset.

## 3. Improved Maximal Cliques for 6D Pose Estimation

The MAC-based pose estimation method models all correspondences as second-order spatially compatible graphs and performs maximal clique searches within these graphs. However, as the number of correspondences increases, the computational efficiency of this method significantly deteriorates. The maximal clique search becomes a performance bottleneck due to its exponential complexity. Moreover, while the MAC method typically relies on the inlier count criterion to select the optimal pose transformation matrix, the accuracy of this criterion decreases when large outliers are present in the point cloud correspondences.

**Figure 4:**
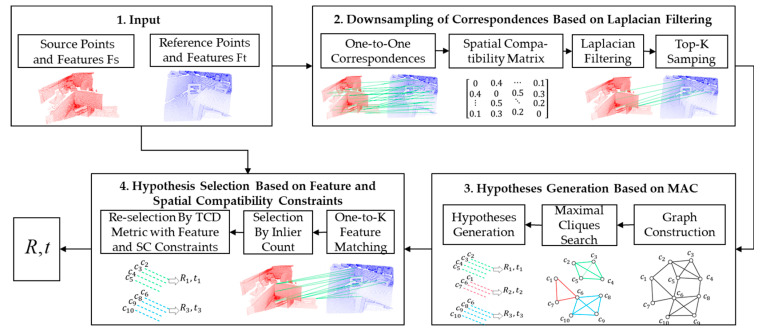
Pipeline of improved MAC.

To address these issues, this paper proposes an improved MAC method, as illustrated in Figure 4. The proposed method comprises three main stages: downsampling of matching pairs using a Laplacian filter, hypothesis generation based on the maximal clique, and hypothesis selection incorporating both feature and spatial consistency constraints. Specifically, given the input source point cloud with its features and the reference point cloud with its features, the method first establishes initial correspondences and constructs a second-order compatibility graph. Subsequently, the Laplacian filter is applied to downsample the graph nodes, thereby effectively reducing the graph’s scale. Next, the graph construction, maximal clique search, and candidate hypothesis generation strategies from the MAC method are employed to generate multiple candidate 6D pose estimation matrices. Finally, the optimal 6D pose estimation matrix is determined using an evaluation strategy that combines feature matching and spatial consistency constraints.

Compared to the MAC method, the proposed algorithm introduces two key improvements. First, it incorporates a Laplacian filter-based downsampling module to significantly reduce the graph’s scale, thereby enhancing the efficiency of the maximal clique search without compromising the performance of the MAC method. Second, it proposes a pose transformation matrix selection strategy that combines feature consistency with spatial constraints, which significantly enhances the evaluation accuracy of candidate hypotheses.

### 3.1. Downsampling of Matching Pairs Based on Graph Laplacian Filtering

Given the source point cloud P and reference point cloud Q, the two point clouds are first downsampled using the voxel downsampling method. Then, a manually designed or learned feature extraction method is applied to the downsampled point clouds to obtain the feature vectors $F_{p}$ for the source point cloud and $F_{q}$ for the reference point cloud, respectively. Feature matching is then performed to establish the initial correspondence $C_{init}=\left\{\left(p_{i},q_{i}\right)\in R^{6}|i=1,\cdots ,n\right\}$.

To reduce the scale of the graph nodes and shorten the MAC search time, this paper proposes a downsampling method based on the fast maximal cliques point cloud registration method [24]. The method uses a Laplacian filter to select spatially consistent correspondences in the graph space, generating a reduced-scale matching correspondence $C_{new}=\left\{\left(p_{i},q_{i}\right)\in R^{6}|i=1,\cdots ,l\right\},\;l<n$. This new correspondence set is then used as input for the MAC method to perform 6D pose estimation. The specific steps are as follows:

First, a first-order spatial compatibility matrix is constructed for the initial correspondences. This matrix is treated as the adjacency matrix of the graph, and its Laplacian L can be expressed as

(10) L=D−SC

where D is the degree matrix of the first-order spatial compatibility matrix, and it is computed as follows:

(11) $$D=\sum_{j}SC_{ij}$$

Second, the Laplacian matrix is used to perform Laplacian filtering on the generalized degree signal of the graph, retaining the high-frequency components. These components are used as the scoring criteria for selecting inlier points. The filtering process can be expressed as

(12) S=LD

Finally, the matching relations are ranked based on the values of S, and the top-k correspondences are selected to form a new set of matching relations.

The proposed method differs from the FastMAC in two main aspects. First, it applies Laplacian filtering to the first-order spatial compatibility matrix rather than the second-order compatibility matrix. Second, it performs sampling by ranking the nodes based on their scores, instead of using polynomial sampling, which accelerates the sampling process.

### 3.2. Hypothesis Selection Based on Feature and Spatial Compatibility Constraints

After the matching pair downsampling module generates a new set of matching relations,$N_{0}$ candidate pose transformation matrices are produced through graph construction and graph search based on the MAC pose estimation method. The goal is to select the optimal pose transformation matrix among the $N_{0}$ candidates. The MAC method typically relies on inlier count as an evaluation criterion. However, this approach has limitations: it depends on assumed correspondences and lacks global alignment information, which becomes particularly problematic in point cloud pose estimation when the overlap between point clouds is low. To address this issue, a Truncated Chamfer Distance (TCD) evaluation criterion based on feature and spatial compatibility constraints [27] is introduced to improve hypothesis selection accuracy.

In the feature space of the source point cloud P and the reference point cloud Q, a relaxed matching matrix, denoted as $H\in R^{n\times k}$, is constructed via feature matching. Each point in the source point cloud can be matched to k points in the reference point cloud. For the matching matrix H, if the feature space of $q_{j}$ is among the top-k neighbors of $p_{i}$, then $H_{ij}=1$; otherwise, $H_{ij}=0$. Specifically,

(13) $$H_{ij}=\left\{\begin{array}{c} 1,\;\;\;\;if\;F_{q_{j}}\in n_{k}\left(F_{p_{j}}\right)\\ 0,\;\;\;\;\;others\;\;\;\;\;\;\;\;\;\;\;\;\;\;\;\;\; \end{array}\right.\;$$

where $F_{q}$ represents the feature of $q_{j}$, $F_{p}$ represents the feature of $p_{i}$, and $n_{k}$ denotes the k-neighborhood.

Using the H matrix as a feature constraint, a truncated chamfer distance evaluation strategy is developed. Based on the relaxed matching matrix H, the nearest neighbors of $p_{i}$ are identified. If $q_{j}$ is found such that the alignment error between the matching pair $\left(p_{i},\;q_{j}\right)$ is below a threshold, and the feature constraint $H_{ij}=1$ is satisfied, then $p_{i}$ and $q_{j}$ are considered successfully aligned. The evaluation criterion can be expressed as follows:

(14) $${Score}_{k}=\sum_{i=1}^{N_{0\;}}I\left(\left(\underset{H_{\mathrm{ij}}=1}{\min}\left\|R_{k}p_{i}+t_{k}-q_{j}\right\|\right)<\eta \right)$$

Thus, the candidate screening process based on TCD, incorporating feature and spatial compatibility constraints, proceeds as follows: First, the MAC method is used to perform a preliminary evaluation of each candidate pose transformation matrix using the internal point counting criterion. The pose transformation matrix with the highest internal point rate is selected for secondary screening. This criterion ensures that the matrix satisfies the spatial compatibility constraint. Then, the feature constraint is applied to refine the TCD evaluation, and the best pose transformation matrix is selected from the remaining candidates.

## 4. Relative Pose Estimation for Autonomous Docking

Traditional eye-in-hand or eye-to-hand robotic systems rely on a known relationship between the camera and the base coordinate system of the AGV. However, in unstructured docking environments, where the relative posture of both the vehicle and the workbench target are continuously changing, this relationship may be unknown. In such cases, traditional eye-in-hand or eye-to-hand systems cannot be applied effectively.

**Figure 5:**
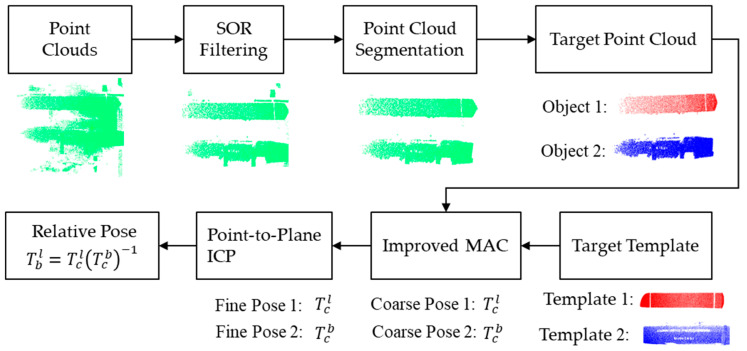
A relative pose estimation framework for automatic docking.

This paper proposes a target relative pose estimation framework for autonomous docking. The framework estimates the relative pose between the vehicle target and the workbench target using the coordinate system of a 3D sensor as an intermediary, thereby guiding the AGV to accurately reach the docking position. The 3D sensor consists of an RGB sensor and a depth sensor, with its coordinate system centered at the depth camera. The coordinate system is defined according to the right-hand rule: the positive X-axis points to the right, the positive Y-axis points downward, and the positive Z-axis points forward, representing the sensor’s measurement range. The proposed framework consists of the following key modules: statistical outlier removal (SOR) filtering, point cloud target segmentation, rough pose estimation based on an improved MAC algorithm, point-to-plane Iterative Closest Point (ICP) fine pose estimation, and relative pose acquisition. The overall process is illustrated in Figure 5.

### 4.1. Removing Outliers by SOR Filtering

In the process of point cloud acquisition, factors such as illumination changes, target movement, and other environmental influences can introduce noise, significantly affecting the accuracy of 6D pose estimation. To mitigate this, the Statistical Outlier Removal (SOR) algorithm is employed for noise filtering. The SOR filter identifies outliers by analyzing the statistical characteristics of the local point neighborhood. The specific process is as follows:

Nearest Neighbor Search: For each point in the point cloud, its k nearest neighbors are identified using the KD-Tree method.

Distance Calculation: The average distance between each point and its k nearest neighbors is computed:

(15) $${\bar{d}}_{i}=\frac{1}{k}\sum_{j=1}^{k}d_{ij}$$

where $d_{ij}$ is the Euclidean distance between point $p_{i}$ and its j-th nearest neighbor, ${\bar{d}}_{i}$ is the average distance between point $p_{i}$ and its k-nearest neighbors, and $p_{i}={\left(x_{i},y_{i},z_{i}\right)}^{T}$ and $p_{j}={\left(x_{j},y_{j},z_{j}\right)}^{T}$ are the coordinates of the  i-th and j-th points in the point cloud, respectively.

Global Mean and Standard Deviation Calculation: The global mean distance μ and standard deviation σ for all points in the point cloud are calculated based on the average distance $\bar{d}$ of each individual point.

Outlier Detection and Removal: A threshold D=μ+λσ, controlled by a constant λ, is defined to determine the outlier criterion. Points whose average distances ${\bar{d}}_{i}$ exceed the threshold D, indicating they are far from their neighbors, are identified as outliers and removed from the point cloud.

### 4.2. Point Cloud Target Segmentation Based on 2D Image Segmentation

Direct target segmentation from point cloud data presents several challenges. Traditional point cloud segmentation methods are often unstable, imprecise, and sensitive to environmental variations. While deep learning-based segmentation techniques offer higher accuracy, they require large amounts of labeled data that can be time-consuming and labor-intensive to annotate, especially for point cloud data. Furthermore, acquiring real labeled data is a difficult task. In contrast, segmentation methods based on 2D images are more convenient for data annotation and training. To address these challenges, this paper proposes a 3D object segmentation approach that leverages 2D image segmentation techniques.

The proposed method simultaneously acquires both point cloud data and RGB images. Subsequently, using the known extrinsic parameters between the 3D sensor and the RGB sensor, all points in the point cloud are projected into the RGB sensor’s coordinate system through a perspective transformation [28]. The projection transformation first performs a coordinate transformation, converting points from the 3D sensor’s coordinate system to the RGB sensor’s coordinate system. The formula is as follows:

(16) $$p^{c}=Rp^{d}+t$$

where $p^{d}={\left(x^{d},y^{d},z^{d}\right)}^{T}$ is the coordinate of a point in the 3D sensor coordinate system, and $p^{c}={\left(x^{c},y^{c},z^{c}\right)}^{T}$ is the corresponding coordinate in the RGB sensor coordinate system.

Next, using the intrinsic sensor parameters K of the RGB sensor, the point cloud in the RGB sensor’s coordinate system is projected onto the 2D image plane. The transformation is given by

(17) $$\left[\begin{array}{c} u\\ v\\ 1 \end{array}\right]=\frac{1}{z^{c}}Kp^{c}$$

where (u, v) are the pixel coordinates in the RGB image.

Through these steps, a mapping relationship between the 3D point cloud and the RGB image pixel coordinates is established. Using this mapping, the EU-Net algorithm [29] is applied to segment the 2D image, which in turn allows for the segmentation of the corresponding 3D target point cloud. This approach enables the segmentation of both vehicle-mounted and workbench targets, providing the 3D segmentation results.

### 4.3. Estimation of Relative Pose of Point Cloud

In the pose estimation process, this paper employs a coarse-to-fine strategy for 6D pose estimation. First, the template point cloud and the segmented target point cloud are input. The improved MAC pose estimation algorithm is then applied for an initial rough estimation. Following this, the point-to-plane Iterative Closest Point (ICP) algorithm is used for fine estimation.

The point-to-plane ICP algorithm [30] operates by iteratively optimizing the transformation matrix between the source point cloud P, the corresponding reference point cloud Q, and the normal vector n, using the following formula:

(18) $$T^{*}=\underset{T}{\mathrm{argmin}}\sum_{i}{\left(\left(T\cdot p_{i}-q_{i}\right)\cdot n_{i}\right)}^{2}$$

In this process, the point-to-plane ICP algorithm uses the result from the improved MAC algorithm as the initial pose, with a maximum distance threshold of 0.01 for corresponding point pairs. The termination condition is either when the average distance of the closest points is less than $10^{-6}$ or when the number of iterations reaches 30.

For the 6D relative pose estimation of the vehicle and workbench targets, the key task is to estimate the rigid body transformation matrix from the target to the sensor coordinate system. Based on the known template point cloud information, the rigid body transformation matrices $T_{c}^{b}$ and $T_{c}^{l}$ for the vehicle and workbench targets can be obtained, respectively. The relative pose between the two targets can then be estimated using Equation (22), thereby facilitating the autonomous docking operation.

(19) $$T_{b}^{l}=T_{c}^{l}{\left(T_{c}^{b}\right)}^{-1}$$

## 5. Experiments

### 5.1. Experimental Configuration

#### 5.1.1. Datasets

This paper evaluates the effectiveness of the proposed improved MAC pose estimation method on three publicly available datasets: 3DMatch, 3DLoMatch, and KITTI. The 3DMatch and 3DLoMatch datasets are scene-scale indoor datasets derived from eight different indoor environments. 3DLoMatch, a subset of 3DMatch, has a low overlap rate of less than 30% between the source and reference point clouds, making it a particularly challenging dataset. The KITTI dataset, an outdoor dataset, is aligned with the MAC algorithm and includes 555 pairs of point clouds used for testing.

**Figure 6:**
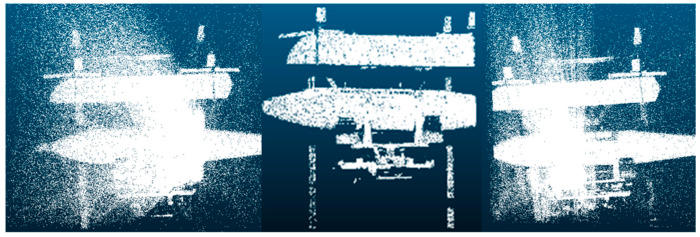
Automatic docking dataset.

To evaluate the proposed relative pose estimation framework for autonomous docking applications, a dedicated autonomous docking dataset was created. The dataset is derived from a simulated aircraft component docking scenario. In this simulation, both the mounted and target components are fabricated using 3D printing technology based on the models of aircraft parts. The mounted component is placed on a four-wheel omni-directional AGV, while the target component is fixed on a workbench. The position of the 3D sensor relative to the docking target is set at varying distances and angles. A total of nine different data points were collected. A subset of the point cloud data is shown in Figure 6, where the true relative pose was accurately estimated using the ICP algorithm after manual matching and labeling.

#### 5.1.2. Evaluation Metrics

In line with the MAC method, the performance of the proposed algorithm on the scenario-scale datasets is evaluated using three metrics: rotation error (RE), translation error (TE), and recall rate (RR).

Rotation error (RE) is defined as follows:

(20) $$RE=\frac{1}{N}\sum_{i=1}^{N}\cos^{-1}\left(\frac{trac\left({\hat{R}}_{i}^{-1}R_{i}\right)-1}{2}\right)$$

where $R_{i}$ is the true rotation matrix between the source and reference point clouds, and ${\hat{R}}_{i}$ is the rotation matrix predicted by the 6D pose estimation algorithm.

Translation error (TE) is defined as follows:

(21) $$TE=\frac{1}{N}\sum_{i=1}^{N}{\left\|{\hat{t}}_{i}-t_{i}\right\|}_{2}$$

where $t_{i}$ is the true translation vector, and ${\hat{t}}_{i}$ is the translation vector predicted by the 6D pose estimation algorithm.

Recall rate (RR) is a commonly used evaluation metric. Following the settings of the MAC method, pose estimation is considered successful if $RE\leq 15^{{}^{\circ}},TE\leq 30\;cm$ is satisfied for the 3DMatch and 3DLoMatch datasets, and $RE\leq 5^{{}^{\circ}},TE\leq 60\;cm$ is satisfied for the KITTI dataset. The recall rate is defined as the ratio of the number of successful pose estimations to the total number of point cloud pairs to be estimated.

(22) $$RR=\frac{N_{success}}{N}$$

#### 5.1.3. Experimental Setup

The experiments were conducted using Open3D [31], PyTorch [32], and igraph [33], and were implemented in Python 3.8. For feature extraction, the FPFH and FCGF descriptor methods were used to generate the initial set of matching correspondences. All experiments were run on a server configured with an Intel Core i7-12700H CPU, an Nvidia Quadro RTX 8000 GPU, and 64 GB of RAM.

### 5.2. Results for 6D Pose Estimation

#### 5.2.1. Results on 3DMatch Dataset

The effectiveness of the proposed method was evaluated on the 3DMatch dataset. For both the source and reference point clouds, a 0.01 m voxel grid was used for downsampling, and feature extraction was performed using both the manually designed FPFH and the learned FCGF descriptors. Table 1 compares the proposed method with several other geometric-based and deep learning methods, including CG-SAC [20], SC2-PCR [22], MAC [23], FastMAC [24], RAN-SAC [18], GC-RANSAC [34], and TEASER++ [35].

As shown in Table 2, the proposed method achieves the highest recall rate, the smallest rotation error, and the smallest translation error when both manually designed and learned feature descriptors are used. The high recall rate indicates that this method successfully estimated the poses of a larger number of point cloud pairs. Specifically, compared to the MAC method, the recall rate improved by 3.82% with the FPFH descriptor and by 1.48% with the FCGF descriptor. Moreover, with the FPFH descriptor, the translation error decreased from 6.73 cm to 6.33 cm, a reduction of 0.47 cm. Similarly, with the FCGF descriptor, the translation error decreased from 6.54 cm to 6.22 cm, a reduction of 0.32 cm. These results demonstrate the effectiveness of the proposed method in pose estimation.

#### 5.2.2. Results on 3DLoMatch Dataset

The proposed method was also tested on the 3DLoMatch dataset, which is characterized by a low overlap rate. Similarly to the previous experiment, FPFH and FCGF descriptors were used to extract point cloud features and generate initial correspondences. The method was compared with other approaches, including PointDSC [19], SC2-PCR [22], MAC [23], FastMAC [24], RANSAC [18], TEASER++ [35], and DGR [36]. Table 2 presents a comparison of registration recall rate (RR), rotation error (RE), and translation error (TE) for various methods.

As shown in Table 2, both FPFH and FCGF descriptors used in this paper achieved the highest recall rates—42.73% and 61.20%, respectively—improving by 1.85% and 2.35% over the MAC method. These results confirm that the proposed method is effective even when the point cloud overlap is low.

#### 5.2.3. Results on KITTI Dataset

We also conducted detailed tests on the outdoor KITTI dataset. Table 3 shows the results of a comparison between the proposed method and other methods, including PointDSC [19], SC2-PCR [22], MAC [23], FastMAC [24], RANSAC [18], TEASER++ [35], and DGR [36].

Although the KITTI dataset consists of outdoor point cloud data, which are characterized by significant sparsity and an uneven distribution of points, the results in Table 3 demonstrate that, compared to the MAC method, the proposed approach achieves a slight decrease in recall rate while significantly reducing both rotational and translational errors under the FPFH and FCGF feature extraction methods. Specifically, the rotational error decreased by 0.11° and 0.02°, while the translational error decreased by 2.28 cm and 5.49 cm, respectively. Notably, under the FPFH feature extraction method, the proposed approach achieves the best performance in both rotational and translational errors to date. Therefore, the pose estimation experiments conducted on both indoor and outdoor scene datasets validate that the improved MAC method exhibits strong generalization ability across different application environments.

#### 5.2.4. Ablation Experiment

An ablation study was conducted to verify the effectiveness of the proposed method by gradually evaluating the resampling module and hypothesis selection strategy on the 3DMatch dataset. Table 4 presents the results, comparing recall rate (RR) and runtime (T) for different configurations. All experiments were implemented in Python and Open3D, with FPFH descriptors used for feature extraction.

As shown in rows 1 to 3 of Table 4, the MAC method is time-consuming, especially as the number of point cloud matching pairs increases, causing exponential growth in runtime. By downsampling the point cloud matching pairs, the proposed method effectively reduces runtime while slightly sacrificing recall rate. Compared to rows 3 and 4 in Table 4, the improved hypothesis selection strategy significantly improves recall rate. These results confirm that the proposed improvements not only reduce runtime but also enhance the overall performance of the pose estimation method.

### 5.3. Relative Pose Estimation Results on the Autonomous Docking Dataset

The performance of the proposed method was further evaluated in an autonomous docking dataset, using point cloud data collected by a 3D depth sensor based on structured light. A voxel grid of 0.01 m was used for downsampling, and FPFH and FCGF descriptors were employed for feature extraction and initial correspondence generation.

In this experiment, the proposed method was compared with the MAC and RANSAC methods, with the results shown in Table 5. The proposed method achieved the best rotation and translation errors in relative pose estimation for autonomous docking, demonstrating superior robustness and accuracy. However, when using the FPFH descriptor, the lack of feature discrimination led to misalignments between the vehicle and workbench targets, resulting in larger pose errors. In contrast, the FCGF descriptor, due to its stronger feature representation, performed better and facilitated better alignment, particularly when using the deep learning-based method.

## 6. Conclusions

To address the challenges of slow search efficiency in the maximal cliques pose estimation algorithm and the inapplicability of assumption selection strategies in low-overlap correspondences, this paper proposes an improved maximal cliques pose estimation method. The method utilizes node downsampling based on Laplacian graph filtering to retain high-frequency signal nodes, thus reducing the graph’s scale. A truncated Chamfer distance evaluation strategy, incorporating feature fusion and spatial compatibility constraints, is introduced to enhance evaluation accuracy in low-overlap point cloud correspondences. Additionally, relaxed feature space constraints are applied to further improve the evaluation precision. Experiments on the 3DMatch, 3DLoMatch, and KITTI datasets demonstrate that the proposed method achieves superior pose estimation performance, ensures algorithm efficiency, and exhibits robust performance in the presence of noisy point clouds and varying overlap rates. Furthermore, based on the improved maximal cliques method, a relative pose estimation framework for autonomous docking is proposed. Using a coarse-to-fine estimation strategy, the poses of vehicle-mounted and workbench targets in the 3D sensor coordinate system are estimated, allowing the relative pose between the two targets to be computed and facilitating autonomous vehicle docking. Experimental results on autonomous docking datasets validate the framework’s efficiency and robustness.

Future work will focus on collecting additional autonomous docking data across various lighting conditions, scenarios, and target types to further optimize the framework’s adaptability to different autonomous docking scenarios.

## Figures and Tables

**Table 1:** Experimental results of different pose estimation methods on 3DMatch dataset.

	FPFH			FCGF		
	RR (%)	RE (°)	TE (cm)	RR (%)	RE (°)	TE (cm)
GC-SAC [20]	78.00	2.40	6.89	87.52	2.42	7.66
SC2-PCR [22]	83.73	2.18	6.70	93.16	2.09	6.51
MAC [23]	83.90	2.11	6.80	93.72	2.02	6.54
FastMAC@0.5 [24]	82.87	2.15	6.73	92.67	2.00	6.47
RANSAC-1M [18]	64.20	4.05	11.35	88.42	3.05	9.42
RANSAC-4M [18]	66.10	3.95	11.03	91.44	2.69	8.38
GC-RANSAC [34]	67.65	2.33	6.81	92.05	2.33	7.11
TEASER++ [35]	75.48	2.48	7.31	85.77	2.73	8.66
Ours	**86.69**	**2.10**	**6.33**	**94.15**	**1.98**	**6.22**

**Table 2:** Experimental results of different pose estimation methods on 3DLoMatch dataset.

	FPFH			FCGF		
	RR (%)	RE (°)	TE (cm)	RR (%)	RE (°)	TE (cm)
PointDSC [19]	20.38	4.04	10.25	56.20	3.87	10.48
SC2-PCR [22]	38.57	4.03	10.31	58.73	3.80	10.44
MAC [23]	40.88	**3.66**	**9.45**	59.85	**3.50**	**9.75**
FastMAC@0.5 [24]	38.46	4.04	10.47	58.23	3.80	10.81
RANSAC-1M [18]	0.67	10.27	15.06	9.77	7.01	14.87
RANSAC-4M [18]	0.45	10.39	20.03	10.44	6.91	15.14
TEASER++ [35]	35.15	4.38	10.96	46.76	4.12	12.89
DGR [36]	19.88	5.07	13.53	43.80	4.17	10.82
Ours	**42.73**	3.85	10.00	**61.20**	3.60	9.92

**Table 3:** Experimental results of different pose estimation methods on KITTI dataset.

	FPFH			FCGF		
	RR (%)	RE (°)	TE (cm)	RR (%)	RE (°)	TE (cm)
PointDSC [19]	98.92	0.38	8.35	97.84	0.33	20.58
SC2-PCR [22]	99.64	0.32	7.23	**98.20**	0.33	20.95
MAC [23]	**99.46**	0.40	8.46	97.84	0.34	19.34
FastMAC@0.5 [24]	97.84	0.41	8.61	97.84	0.36	**7.98**
RANSAC [18]	74.41	1.55	30.20	80.36	0.73	26.79
TEASER++ [35]	91.17	1.03	17.98	94.96	0.38	13.69
DGR [36]	77.12	1.64	33.10	96.90	0.34	21.70
Ours	99.10	**0.29**	**6.18**	97.12	**0.32**	13.85

**Table 4:** Ablation experiment on 3DMatch. GC: Graph construction. MAC: Maximal cliques. IC: Inlier count. FS-TCD: Truncated chamfer distance evaluation with feature and spatial compatibility constraints.

	RR (%)	T (s)
MAC (GC + MAC Search + IC)	83.90	3.08
Degree signal ordering sampling 50% + GC + MAC Search + IC	82.13	0.88
Laplace filter sampling 50% + GC + MAC Search + IC	82.37	0.90
Laplace filter sampling 50% + GC + MAC Search + FS-TCD	**86.69**	0.91

**Table 5:** Experimental results of different pose estimation methods on automatic docking dataset.

	FPFH		FCGF	
	RE (°)	TE (cm)	RE (°)	TE (cm)
RANSAC + Point-to-plane ICP	134.47	207.37	4.17	19.45
MAC + Point-to-plane ICP	7.86	63.67	0.97	6.21
Ours	**6.7**	**54.25**	**0.96**	**5.82**

## Data Availability

The datasets are publicly available as the maximal cliques datasets (https://pan.baidu.com/s/1KZS2LccseYJZWMmDG0xDbw, password: 1234), accessed on 30 December 2024.
